# 897. Patients with Mpox, Minnesota, 2022

**DOI:** 10.1093/ofid/ofad500.942

**Published:** 2023-11-27

**Authors:** Nicholas Lehnertz, Marcie Babcock, Ali Ruprecht, Jayne Griffith, Ruth Lynfield

**Affiliations:** Minnesota Department of Health, St. Paul, Minnesota; Minnesota Department of Health, St. Paul, Minnesota; Minnesota Department of Health, St. Paul, Minnesota; Minnesota Department of Health, St. Paul, Minnesota; Minnesota Department of Health, St. Paul, Minnesota

## Abstract

**Background:**

A global outbreak of mpox occurred in 2022; we evaluated characteristics of mpox patients in Minnesota.

**Methods:**

Cases of mpox are reportable to the MN Department of Health (MDH). Cases are interviewed to collect demographic, exposure and clinical information; medical records from hospitalized patients (HP) are reviewed to collect data on clinical course, testing and treatment.

**Results:**

Between June 25 and November 5, 2022, 234 lab-confirmed cases of mpox, including 10 HP and 0 deaths were reported; 85% cases were interviewed. Overall median age was 34 years (IQR 29-43), 59% White, 92.3% male and 34% persons living with HIV (PWH). One outpatient received JYNNEOS vaccine > 14 days prior to symptoms; no HP received vaccine. Unstable housing and substance use were reported in 5 and 7 HP respectively. HP, if PWH were 4.1 times more likely to have CD4< 200 or detectable virus (p=0.035). Reasons for hospitalization included oropharyngeal pain/airway compromise (4), painful proctitis (1), isolation (2), epididymitis/orchitis (1), and preseptal cellulitis (1). The median length of initial stay was 4.5 days (range 1-10). A case with concomitant GAS pharyngitis (not PWH) required intubation and prolonged stay. Other longer length of stay occurred for isolation (2 PWH) and for PWH who had non-suppressed HIV and severe mpox disease.

Among HP, mean orthopox testing occurred 6.1 days (range 0-14) and mean tecovirimat initiation 8.6 days (range 3-18), following symptom onset; similar to outpatient cases. Three HP received no mpox therapies (including 1 PWH). Of 5 HP and PWH, 3 had CD4 < 100; all received tecovirimat. One PWH (with CD4 36) required 3 hospitalizations totaling 69 days and multiple courses of po (108 days) and IV (26 days) tecovirimat, VIGIV (6), brincidofivir (4), cidofovir (7), and topical and intralesional cidofovir.

**Conclusion:**

Approximately 4% of reported mpox were HP; reasons varied including unstable housing and need for isolation. Very severe courses occurred in a patient with GAS superinfection and 1 of 3 PWH with low CD4. Attention to potential superinfection, HIV infection and suppression are important. Efforts must be strengthened to reach at risk populations, including those with less access to care, for education and mpox vaccination.

**Disclosures:**

**All Authors**: No reported disclosures

